# Prediction of cell position using single-cell transcriptomic data: an iterative procedure

**DOI:** 10.12688/f1000research.20715.2

**Published:** 2020-04-09

**Authors:** Andrés M. Alonso, Alejandra Carrea, Luis Diambra

**Affiliations:** 1 CREG-CONICET, Universidad Nacional de La Plata, La Plata, Buenos Aires, 1900, Argentina; 2 INTech-CONICET, Universidad Nacional de San Martin, Chascomus, Buenos Aires, Argentina

**Keywords:** Single-Cell RNA sequencing, Drosophila Embryo, Gene expression Patterns, DREAM Challenge

## Abstract

Single-cell sequencing reveals cellular heterogeneity but not cell localization. However, by combining single-cell transcriptomic data with a reference atlas of a small set of genes, it would be possible to predict the position of individual cells and reconstruct the spatial expression profile of thousands of genes reported in the single-cell study. With the purpose of developing new algorithms, the Dialogue for Reverse Engineering Assessments and Methods (DREAM) consortium organized a crowd-sourced competition known as DREAM Single Cell Transcriptomics Challenge (SCTC). Within this context, we describe here our proposed procedures for adequate reference genes selection, and an iterative procedure to predict spatial expression profile of other genes.

## Introduction

Multicellular organisms show throughout their development a crescent cellular heterogeneity, distributed and organized in different organs and tissues. This spatial heterogeneity has been explored using different techniques, such as immunohistochemistry and single-molecule fluorescence *in situ* hybridization (FISH) ^1^. These approaches allow quantification of gene expression in many cells but, unfortunately, these techniques can currently be assayed only over a small number of genes. The selection of these genes introduces a bias that limits the power of these studies. With the advent of emergent methods in genomics, it has become possible to assess the transcriptomic profile of complex tissues with unprecedented resolution, thereby allowing insights into complex processes such as: differentiation trajectories, cell fate decisions, and spatial relationships. In this sense, high-throughput single-cell RNA-seq (sc-RNA-seq) is becoming an established experimental technique ^2^. The protocol of this technique includes the initial step of sample collection, during which solid tissue dissociation results in single cells. Removing cells from their native context results in the loss of spatial information. However, this information can be crucial when the goal is to study the molecular composition of individual cells in the context of spatial location, for example, in the context of primary cancer cells research ^3^. Fortunately, some progress has been made to overcome limitations of spatial information loss associated to this technique. Computational methods, based on Principal Component Analysis, are able to partially recover the spatial structure of gene expression patterns ^4^. More recently, several computational techniques coupled to *in situ* RNA patterns facilitate this reconstruction with better resolution ^5– 7^.

In order to catalyze research on computational methods for the spatial reconstruction of single-cell gene expression data, a crowd-sourced competition was designed by the DREAM Consortium in collaboration with Nikos Karaiskos and Nikolaus Rajewsky from Max Delbruck Institute. Using sc-RNA-seq data from Rajewsky Lab, published in 7, and the expression patterns of driver genes as an expression reference atlas, three main subchallenges were designed. The particular aim was to predict the position of 1297 cells in the 3039 *Drosophila melanogaster* embryonic locations, or bins, for one half of an embryo in stage 6 (pre-gastrulation), based on scR-NAseq data. The prediction of the 1297 cell positions must be done using a limited number of genes selected from a pool of 84 expression patterns used as a reference atlas. In subchallenge 1 the prediction must be performed using 60 driver genes out of 84 genes, in subchallenge 2 using a subset of any expression patterns from 40 genes out of the 84, and in subchallenge 3 using a subset of any expression patterns from only 20 driver genes. The selection of the subset of genes used for the prediction poses an additional and interesting problem. In this paper we present a procedure for solving the cell-position problem posed in the DREAM SCTC. This challenge consists of predicting the positions of individual cells, based on an expression reference atlas and a small set of genes reported in single-cell studies.

## Methods

### DREAM challenge data

Expression patterns used as a reference atlas correspond to 84 driver genes obtained from *in situ* hybridization experiments; the data correspond to The Berkeley Drosophila Transcription Network Project (BDTNP) ^8^. This gene expression data set is listed in the file bdtnp.csv at DVEX server. One half of the *Drosophila* embryo has 3039 cells locations, each location is specified by three coordinates ( *x*, *y* and *z*) (geometry.txt at DVEX). Thus, the reference database consists of an expression matrix of 84 genes (columns) quantified across the 3039 embryonic locations (rows). These data were next binarized ^7^, sorted in the same order of cell location, and listed in an additional file (binarized_bdtnp.csv at DVEX server). The single-cell RNA sequencing data is provided as a matrix with 8924 genes as rows, and 1297 cells as columns. These data are divided by the total number of counts for that cell, in this step a pseudocount is added. The normalized values are obtained by taking the logarithm of the total counts. The normalized values are also binarized, i.e. a given gene is ON (OFF) if the normalized values are above (below) of a quantile value. Based on a distance minimization criterion, the quantile value was chosen as 0.23. The short sequences for each of the 1297 cells in the raw and normalized data are the barcodes of individual cells. Both normalized as well as binarized data were provided by the DREAM Challenge.

### Selection of the gene sets

In order to select the gene sets to be used in each subchallenge, we take into account two criteria:

(i) Genes that have complementary expression patterns across the single-cell population. It is well known that many genes are co-expressed, that is, their expression profiles are highly correlated. This correlation introduces a degree of redundancy in the expression matrix, which frequently is reduced by clustering those genes with similar expression profiles. This step allows us to identify genes with complementary expression patterns.
(ii) Genes with expression levels broadly distributed across the single-cell population. This step is performed in order to select one gene per cluster. Those genes with many null expression values over a large part of the population are discarded, because they are associated with distributions with a large peak at zero.

**Figure 1.**
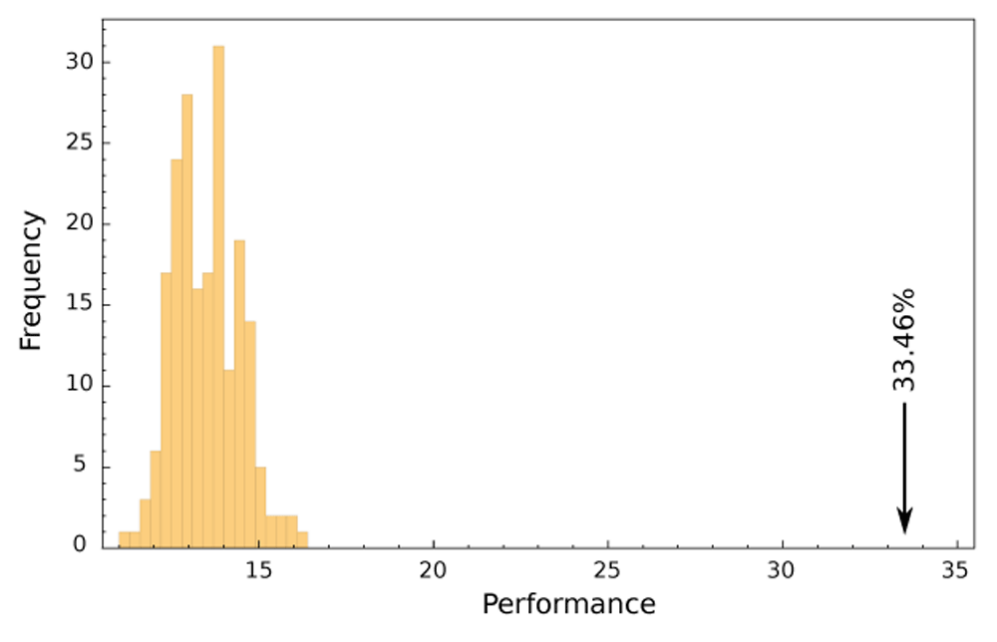
Performance of the gene selection procedure. Histogram of the performance obtained with 20 genes selected at random (yellow).The performance obtained with the set of 20 genes selected by the proposed method is indicated with a black arrow.

To accomplish these criteria, we first perform an agglomerative clustering procedure over the expression matrix comprising the 84 genes (the same genes as the available in the *in situ* expression data) over the 1297 cells). We cluster genes with similar expression profiles across the cells, by means of using the Euclidean distance over the normalized gene expression levels, and the Unweighted Pair Group Method with Arithmetic Mean (UPGMA) as a linkage method. Then, we cut the dendogram tree into 20, 40, or 60 groups depending on the subchallenge. Next, we need to select only one gene per cluster. This selection is performed based on the criterion of the broadest distribution. To this end, for each gene within a given cluster we compute the frequency distribution *p _i_*, where *p _i_* denotes the frequency of occurrence of expression levels within the bin *i*. Here, we set the bin size equal to 0.125. After that, we compute the associated entropy $H\;=\;–\;\sum_{i}p_{i}\;1n\;p_{i}$. Then, we select the gene with the greatest entropy in each cluster, i.e. the gene within the cluster with the broadest expression distribution across the single-cell population. This selection procedure is performed with the R script named preprocessing.r, which uses the function selgen.R, both available at Zenodo (see *Data availability*). To assess this method for the gene selection, we compare the prediction performance obtained with the set of 20 genes selected in this way with the results obtained with different sets of genes sampled at random. For comparison, we consider the Mathews correlation coefficient (MCC) ^10^ between the 1297 cells and the 3039 bins. Then, the ten better scored bins are selected as putative position for each cell. As the true positions of the cells are not available, we take the bin with the highest MCC, obtained with the set that include all 84 genes, as the bin associated with the true position. Thus, we count cells with the ten best scores containing the true position as cells whose positions are well predicted. The percentage of the well-predicted positions will be our measure of the performance. Figure 1 depicts the histogram of percentage of cells with well-predicted positions, obtained with 200 sets of 20 randomly selected genes. In all cases, this percentage is quite lower than that obtained with 20 genes selected as indicated above, which is 33.46%.

We use this procedure to select an additional set of 100 genes from the 8924 genes measured by the single-cell technique, but excluding the genes from the 84 reference gene set. These 100 genes will be used in further steps during the iterative procedure, and will be denoted as the outgroup set hereafter. The 20, 40 and 60 selected genes used for each cell location prediction task are listed in Table S1 (see *Extended data*); we also include the outgroup set of genes.

### Scoring functions

**Figure 2.**
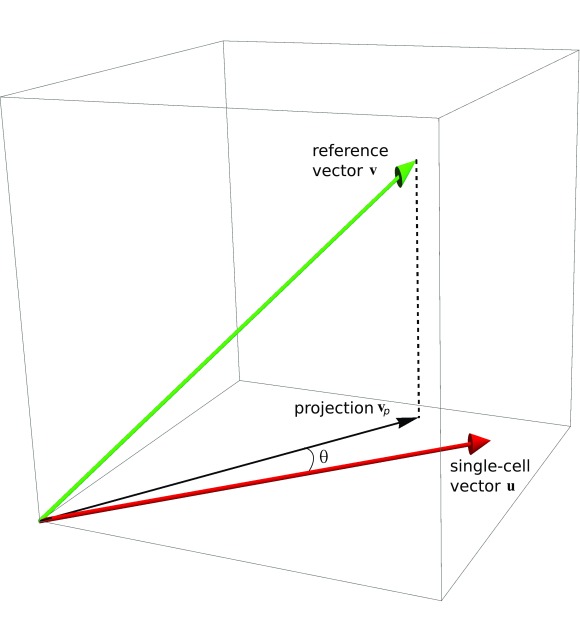
Overlap-based score. Low-dimensional representation of the angle between the expression vector **u**, and the projected expression vector **v** *_p_*.

In order to predict the position of a given single cell, we use a score approach based on two similarity measures between the sc-RNA-seq data, and the reference atlas. One of these measures is the MCC computed between the binarized expression profiles, as proposed in 7. The MCC will be used in the initial step to assign putative bin positions for each single cell, and then to predict the spatial expression profile of the outgroup set of genes. The other measure is the overlap between the normalized expression vector of the single cells, and the projected vector corresponding to the predicted spatial expression profile. This vectorial space corresponds to the one spanned by the outgroup set of genes only. The overlap is defined by: $\text{cos}(\theta )=\frac{\text{u}\cdot {\text{v}}_{p}}{\text{u}{\text{v}}_{p}},$ where **u** is the profile vector of the single cell, and **v**_*p*_ is the vector obtained by projecting the profile vector of the predicted profile on the subspace spanned by the non-null components of the profile vector **u**, as illustrated in Figure 2. The scoring functions are performed by the R script named functions.r, available at Zenodo (see *Data availability*).

## Results

**Figure 3.**
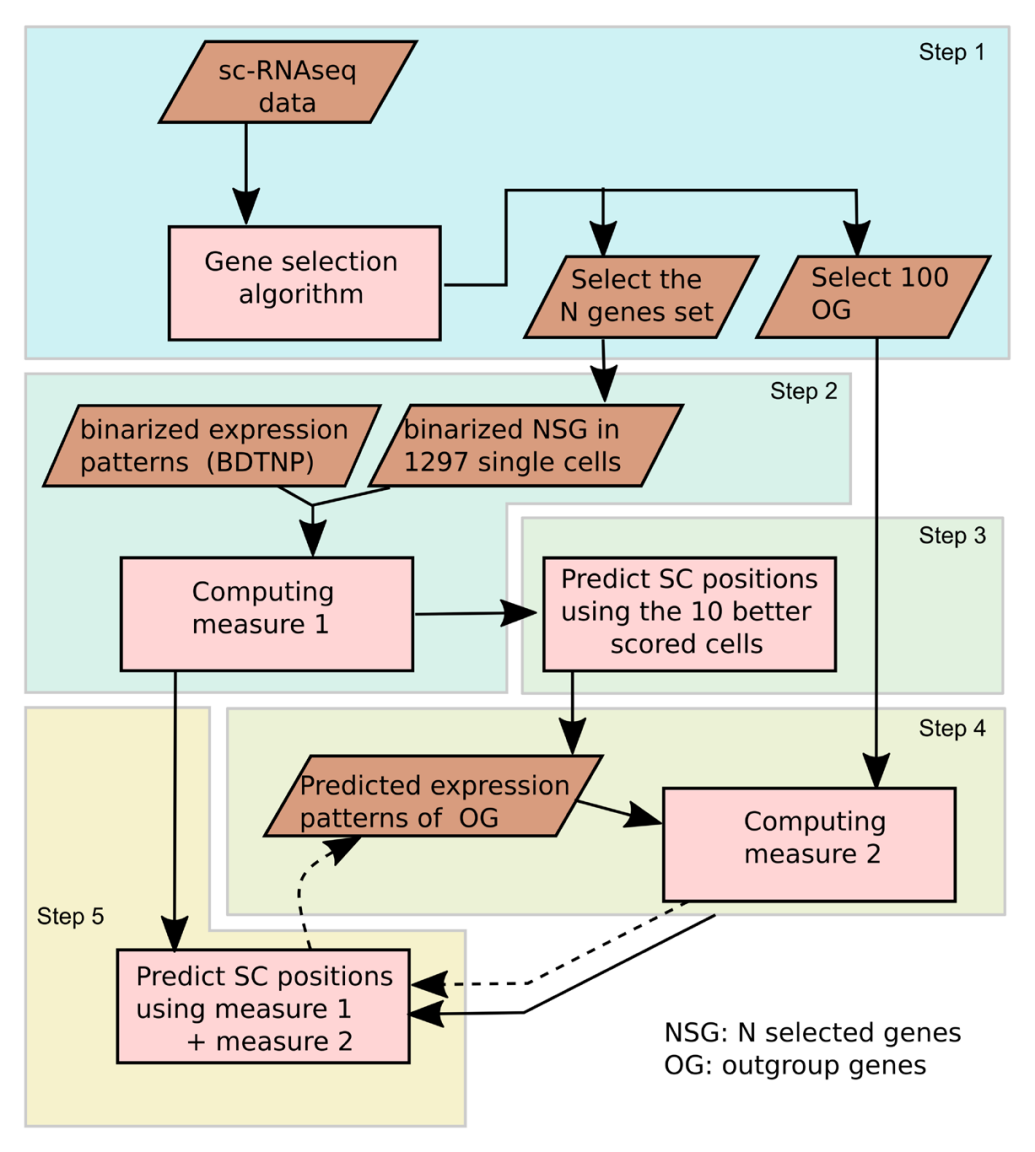
Flow diagram of the proposed method. Step 1: The set of *N* genes and the additional 100 outgroup genes are selected from the sc-RNAseq data. Step 2: Using the binarized expression data of the *N* selected genes we compute measure 1 for the1297 single-cell vectors against the 3039 binarized vectors of the reference atlas. Step 3: We predict the single-cell positions using the positions of the 10 better scored cells. Step 4: We build the putative expression patterns of the outgroup set of genes and we compute measure 2 against the expression level of 1297 single-cells. Step 5: By means of using the composed score *S*, the predicted expression patterns of the outgroup set of genes is improved in each iteration. The last two steps are repeated (2 or 3 times).

The proposed procedure is schematically illustrated in Figure 3. In the first step we select the set of *N* genes from the 84 driver genes to be used in the prediction, using the method described in *Selection of the gene sets* section. We also select an additional 100 genes (outgroup set of genes) from all genes measured in the sc-RNAseq experiment, but excluding the driver genes. The name of the genes used are listed in *Extended data*: Table S1. Then, using the binarized expression data of the selected genes, we compute the MCC (measure 1) for each binarized single-cell vector against the 3039 binarized vectors associated with each positional bin of the reference atlas (BDTNP). By means of the MCC-based score, we predict the single-cell positions and build the putative expression patterns of the outgroup set of genes. In this sense, the expression level of gen *g* at the bin position *i* is given by the weighted average of the normalized gene expression across 10 putative positions corresponding to that bin, being the weight proportional to the associated MCC. Mathematically, $e_{i}^{g}=\sum_{j}^{*}c_{ij}\;e_{j}^{g},$ where *c _ij_* are the MCC-based scores of the single cell *j* against position *i*, and $e_{i}^{g}$ are the expression levels of gene *g* recorded in the individual cells *j*. The asterisk in the summation indicates that the 10 first better scored cells positions are included. The predicted expression patterns of the outgroup set of genes computed in this manner are used to compute the overlap (measure 2) with the corresponding expression level of each one of the 1297 single cells. Finally, using the measure 1 and the measure 2 we compute a composed score *S*, defined as *S* = *w*_1_ ∗ *c* + *w*_2_ ∗ *o*, where *c* is MCC-based score, *o* is the overlap-based score, and *w*_1_ and *w*_2_ are the respective weights. The score *S* is used to predict positions and improve the predicted expression patterns of the outgroup set of genes in each iteration. The last two steps are repeated (2 or 3 times), as indicated in Figure 3 with dashed arrows.

**Figure 4.**
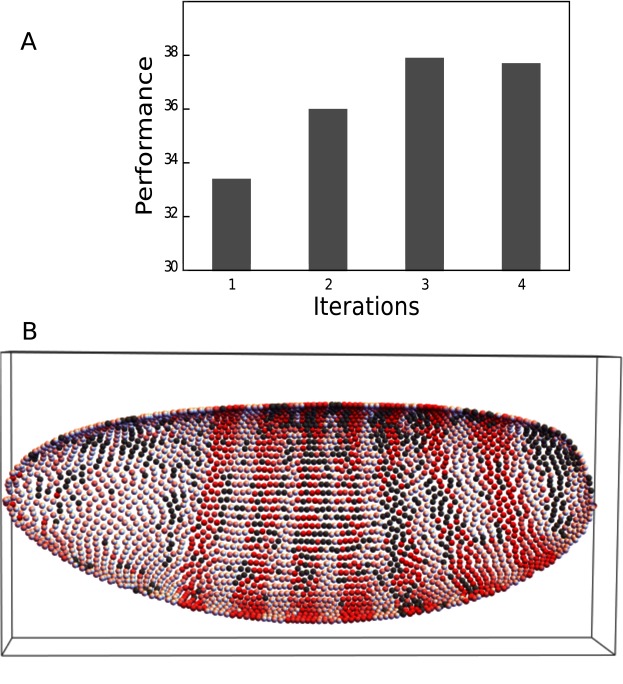
Prediction performance. Panel **A**: Performance obtained by means of using the iterative procedure with 20 genes. Panel **B**: Predicted expression pattern of the *ftz* gene obtained with 60 genes after two iteration steps. The expression level of each nuclei is given in white-red scale. Gray nuclei correspond to positional bins without prediction.

The above scheme is applied to the subchallenges with 20, 40 and 60 genes using different weight values. First of all, we apply the procedure to the subchallenge 3. Using the 20 genes we compute the MCC for every cell-bin combination. The first iteration of this scoring procedure leads to a performance of 33.5% in assigning the putative positions to each single cell. By means of using the 20 highest coefficients, we predict the expression patterns of the outgroup set of genes. Then, we compute a scoring measure composed of two terms: the MCC computed in the first step (with a weight *w*_1_ = 0.7), and the previously-defined overlap between the expression vector of each single cell and the projected expression vector of the reference atlas, being both vectors composed of the 100 outgroup genes (with a weight *w*_2_ = 0.3). The score combining both measures is then used to predict the positions of each single cell, which leads to a performance of 36% in the second iteration, and 38% in the third iteration. Further iteration steps do not produce any additional improvement. Figure 4A depicts the performance evolution of the procedure using this gene set.

In order to select the set of 60 genes to be used in subchallenge 1, from the 84 genes available in the reference atlas, we perform the above-mentioned agglomerative clustering procedure. Then, the 60 genes with the greatest entropy within each cluster are selected. The names of the resulting genes are listed in the first column of *Extended data*: Table S1. As a first step, we compute the MCC for each binarized single-cell vector, and the corresponding 3039 binarized vectors associated with each positional bin of the reference atlas. By means of using the 20 highest MCC for each cell ( *N* = 20), we compute the putative expression patterns of the outgroup set of genes. In this case, the used scoring measure was composed by MCC with a weight of 0.90; and the overlap of the single-cell expression profiles and the 3039 positions of the predicted expression patterns obtained in the previous step, with a weight of 0.10. After two iterations the performance obtained is 95.4%. Figure 4B shows the predicted expression pattern of the *ftz* gene obtained using the set of 60 genes. The same procedure is used to predict the positions of single cells by considering a set of 40 genes. Again, these genes are selected as described in Methods. The names of the resulting genes are listed in the second column of *Extended data*: Table S1. In this case, the performance obtained reaches 71.4%.

## Discussion

We present three innovations that could represent improvements in regard to the original proposal ^7^. One of these innovations is the method for selecting the set of genes to be used as reference in the cell-positions prediction task. This set of genes is a good starting point in the presented strategy for position prediction, although we have not explored this method in depth. For example, the Jaccard distance 10 could be used in the clustering procedure instead of the Euclidean distance. We noticed that MCC can overestimate false negatives due to the fact that sc-RNA-seq are not able to record expression of many genes. This results in profiles with many zeros, even in cases of moderate expression levels. For that reason, our second proposed innovation is an alternative way to make the comparison between profiles, as we used in subchallenge 3. Last but not least, the third innovation is the iterative procedure, which improves the performance of any of the alternative strategies presented here. In addition, we noticed that the iterative procedure does not necessarily converge to the correct solution, may be due to error propagation on the predicted patterns.

## Data availability

### Underlying data

Challenge documentation, including the detailed description of the Challenge design, overall results, scoring scripts, can be found at: http://www.synapse.org/#!Synapse:syn15665609/wiki. Data for this Challenge can be downloaded from http://shiny.mdc-berlin.de/DVEX/.

Zenodo: Prediction of cell position using single-cell transcriptomic data: an iterative procedure, https://doi.org/10.5281/zenodo.3470061 ^11^.

This project contains code and documentation underlying the methods.

Data are available under the terms of the Creative Commons Zero "No rights reserved" data waiver (CC0 1.0 Public domain dedication).

### Extended data

Zenodo: Prediction of cell position using single-cell transcriptomic data: an iterative procedure, https://doi.org/10.5281/zenodo.3470061 ^11^.

This project contains the following extended data:

- Table S1: **Selected genes:** first, second and third columns list the name of genes used in the subchallenges 1, 2 and 3, respectively. The last column lists the names of the outgroup genes.

Data are available under the terms of the Creative Commons Zero "No rights reserved" data waiver (CC0 1.0 Public domain dedication).
