# Supplementary material for: Prediction of cell position using single-cell transcriptomic data: an iterative procedure
Source: F1000Res. 2020 Apr 9;8:1775. Originally published 2019 Oct 18. [Version 2] doi: 10.12688/f1000research.20715.2 (PMC7194340; doi:10.12688/f1000research.20715.2)
Supplement: Peer review history

# Peer review: Reviewer response for version 2

**DOI:** 10.5256/f1000research.25587.r62174

**Published:** 2020-04-14

**Authors:** Pablo Meyer

**Affiliations:** 1 IBM research Computational Biology Center, New York, NY, USA

Authors have addressed my concerns.

Is the rationale for developing the new method (or application) clearly explained?

Yes

Is the description of the method technically sound?

Yes

Are the conclusions about the method and its performance adequately supported by the findings presented in the article?

Yes

If any results are presented, are all the source data underlying the results available to ensure full reproducibility?

Yes

Are sufficient details provided to allow replication of the method development and its use by others?

Yes

Reviewer Expertise:

Computational biology and systems biology

I confirm that I have read this submission and believe that I have an appropriate level of expertise to confirm that it is of an acceptable scientific standard.

# Peer review: Reviewer response for version 2

**DOI:** 10.5256/f1000research.25587.r62173

**Published:** 2020-04-14

**Authors:** Roosevelt Alves Da Silva

**Affiliations:** 1 Física, Universidade Federal de Goiás, Jataí, Brazil

All requested revisions have been made. The article is now in the format suitable for the reader.

Is the rationale for developing the new method (or application) clearly explained?

Yes

Is the description of the method technically sound?

Yes

Are the conclusions about the method and its performance adequately supported by the findings presented in the article?

Yes

If any results are presented, are all the source data underlying the results available to ensure full reproducibility?

Yes

Are sufficient details provided to allow replication of the method development and its use by others?

Yes

Reviewer Expertise:

Biological physics, computational science, protein structures.

I confirm that I have read this submission and believe that I have an appropriate level of expertise to confirm that it is of an acceptable scientific standard.

# Peer review: Reviewer response for version 1

**DOI:** 10.5256/f1000research.22783.r55478

**Published:** 2019-11-08

**Authors:** Pablo Meyer

**Affiliations:** 1 IBM research Computational Biology Center, New York, NY, USA

Alonso *et al* propose a nice idea for solving the gene to position problem posed in the DREAM single cell transcriptomics challenge, but at the end fall short while finding ways to improve their pipeline. The explanations of why they used such approach are also scarce. Although maybe obvious, it would be important that they explain why they chose genes with maximum entropy. why 100 outgroup genes, how the values of w1 and w2  were selected and  why doing a 2 step optimization. Overall the rationale of their approach should be summarized in a couple of sentences in order to make it clearer, unfortunately the diagram does not clarify the rationale either. Also, given that this criteria did not improve significantly while iterating, they should have tried other selection criteria such as high expression level, or maybe other metrics such as the ones proposed in the challenge. Maybe trying to optimize the position and the gene expression correlation as interlocked steps is the source of the problem. Some discussion regarding this should be added.

Is the rationale for developing the new method (or application) clearly explained?

Yes

Is the description of the method technically sound?

Yes

Are the conclusions about the method and its performance adequately supported by the findings presented in the article?

Yes

If any results are presented, are all the source data underlying the results available to ensure full reproducibility?

Yes

Are sufficient details provided to allow replication of the method development and its use by others?

Yes

Reviewer Expertise:

Computational biology and systems biology

I confirm that I have read this submission and believe that I have an appropriate level of expertise to confirm that it is of an acceptable scientific standard, however I have significant reservations, as outlined above.

# Response

**Published:** 2020-03-31

**Authors:** Luis Diambra

**Affiliations:** Centro Regional de Estudio Genomicos, Argentina

Alonso et al propose a nice idea for solving the gene to position problem posed in the DREAM single cell transcriptomics challenge, but at the end fall short while finding ways to improve their pipeline. 

**Response: **We would like to thank the Referee 2 for reviewing our paper, and for his positive comments. Below we gives him a point by point response for all his comments.

 The explanations of why they used such approach are also scarce. Although maybe obvious, 

 it would be important that they explain why they chose genes with maximum entropy.

  why 100 outgroup genes,

  how the values of w1 and w2  were selected 

  and  why doing a 2 step optimization. 

**Response:** The approach for our gene set selection has two steps. We agree that these steps have not been justified in our previous version. Now we have added a rationale for our procedure. It is well known that many genes co-express, that is, their expression profiles are highly correlated. This correlation introduces a degree of redundancy in the expression matrix, which is oftenly reduced by clustering those genes with similar expression profiles. We assume that the genes within each cluster would not provide much more information than just one in this group. Since the challenge requires choosing a small set of genes, we must choose one gene that represents each cluster. The question is then, which one? 

 This selection is made with the second criterion, which discards those genes with many null values of expression over a large part of the population, because they are associated with distributions with a large peak at zero. Of course, one could also use a criterion that selects the gene with higher expression level, as you proposed (see last response). Regarding the number of genes used as the outgroup genes, we did not make any computation to determine this number, or to answer if there is an optimal number of genes.  We just chose a number of genes greater than the 86 genes, which seems to be good enough for the correct prediction of the cell positions. 

 Regarding the values of w1 and w2, unfortunately we cannot imagine other method to determine w1 and w2 different from the brute force. In this sense, we began our study with a value of 0.5 for each weight. That gave quite bad results. Then, we increased the weight w1 and decreased w2 by 0.10 at each step, obteining improvements in the performance, until we reached the weights shown in the paper. We did not try to find the better weitghs. Initially, we assumed that an iterative procedure could be a kind of self-consistent method that would lead to an improvement in performance at every step. Unfortunately, that was not verified. The iterative procedure improved the performance only in the first steps. For this resubmission, we have made several modifications and performed perdictions, testing other hypotheses. However, we did not manage to substantially improve our own results (see the last point). 

 Overall the rationale of their approach should be summarized in a couple of sentences in order to make it clearer, unfortunately the diagram does not clarify the rationale either. 

**Response:** We have remade the diagram of Fig. 3, and we have added a short description to each step in the legend of this figure.

 Also, given that this criteria did not improve significantly while iterating, they should have tried other selection criteria such as high expression level, or maybe other metrics such as the ones proposed in the challenge. Maybe trying to optimize the position and the gene expression correlation as interlocked steps is the source of the problem. Some discussion regarding this should be added. 

**Response:** We have evaluated the performance of the approach by considering several modifications. In particular, those proposed by the referee:

 1.- We selected the genes with higher expression levels within each cluster, instead of the genes with broadest level 

 distribution. This gene selection strategy gave similar results to the one presented in the original version.

 2.- We used the metrics based only on MCC, instead of the combination of two metrics. 

 In this case, the predicted expression patterns computed in the first iteration are used to compute the MCC of the of the outgroup genes with the corresponding expression level of each one of the 1297 single cells. Finally, we computed a composed score S, defined as S = w1* c + w2* o, 

 where c is MCC score computed in the first step (with the 20 genes), and o is the MCC score computed 

 with the outgroup set of genes, and w1 and w2 are the respective weights. We do not understand the reasons why subsequent iterations do not improve performance, significantly. However, we believe that the predicted expression pattern of the outgroup set of genes could be used to improve, in subsequent steps, any other method with better performance than the one we have proposed.

# Peer review: Reviewer response for version 1

**DOI:** 10.5256/f1000research.22783.r55476

**Published:** 2019-10-24

**Authors:** Roosevelt Alves Da Silva

**Affiliations:** 1 Física, Universidade Federal de Goiás, Jataí, Brazil

In their manuscript entitled “Prediction of cell position using single-cell transcriptomic data: an iterative procedure ” Alonso *et al.* apply a method to predict the cell position combining single-cell transcriptomic data with a reference atlas. The study is submitted as part of the SCTC DREAM Challenge, which aims to evaluate different algorithms for this prediction task using 20, 40 or 60 genes as reference. The authors test their approach on SCTC challenge data-sets. They have three improvements on a method previously published (ref 7 ^1^): gene selection, a new scoring function, and an iterative procedure for improve the predictions. The paper is generally well-written, with a concise description of the problem and challenge. However it has room for improvement, especially in the manner information is presented. My criticisms are related to the clarification of details I thought to be missing from Methods and Results sections. 

**Major comments:**

1. I suggest to authors to add a sentence that help to reader to understand the aim of the paper in the Introduction.
2. The fluxogram of Fig.3 seems has not a initial point. Why? I suggest to authors divide the procedure in layers. Also authors must give a description in the legend of this figure.
3. Panel A of Fig. 4 has not legend, please add one.

**Minor comments:**

1. The description of acronyms MCC is duplicated. The author must introduce it in the first time that it is mentioned.
2. Please, add adequate references for Mathews correlation coefficient (in Methods) and for Jaccard distance (in Discussion).

Is the rationale for developing the new method (or application) clearly explained?

Yes

Is the description of the method technically sound?

Yes

Are the conclusions about the method and its performance adequately supported by the findings presented in the article?

Yes

If any results are presented, are all the source data underlying the results available to ensure full reproducibility?

Yes

Are sufficient details provided to allow replication of the method development and its use by others?

Yes

Reviewer Expertise:

Biological physics, computational science, protein structures.

I confirm that I have read this submission and believe that I have an appropriate level of expertise to confirm that it is of an acceptable scientific standard, however I have significant reservations, as outlined above.

## References

[1] : TheDrosophila embryo at single-cell transcriptome resolution. *Science*.2017;358(6360) : 10.1126/science.aan3235 194-199 10.1126/science.aan3235 28860209

# Response

**Published:** 2020-03-31

**Authors:** Luis Diambra

**Affiliations:** Centro Regional de Estudio Genomicos, Argentina

**Response:** We would like to thank Referee 1 for reviewing our paper, and for his/her positive comments.

 Below, we give him/her a point by point response for all his/her comments.

 Major comments:

 I suggest to authors to add a sentence that help to reader to understand the aim of the paper in the Introduction. 

 Response: We agree. We have added two sentences at the end of the Introduction in order to clarify the aim of our work.

     

 The fluxogram of Fig.3 seems has not a initial point. Why? I suggest to authors divide the procedure in layers. Also authors must give a description in the legend of this figure.

**Response:** In this new version of the manuscript we have added a description to Fig. 3, and modified its initial point and the layers in order to make them clearer.

      

 Panel A of Fig. 4 has not legend, please add one. 

**Response:** In this new version of the manuscript we have added a legend to Fig. 4A.

 Minor comments:

 The description of acronyms MCC is duplicated. The author must introduce it in the first time that it is mentioned. 

**Response:** The referee is correct, we have already fixed it. 

      

 Please, add adequate references for Mathews correlation coefficient (in Methods) 

 and for Jaccard distance (in Discussion). 

**Response:** We have added two new references: ref10: 10.1016/0005-2795(75)90109-9 and ref11:

 10.1038/234034a0, respectively.
